# Analysis of the Variation in Antioxidant Activity and Chemical Composition upon the Repeated Thermal Treatment of the By-Product of the Red Ginseng Manufacturing Process

**DOI:** 10.3390/molecules29133092

**Published:** 2024-06-28

**Authors:** Yu-Dan Wang, Hui-E Zhang, Lu-Sheng Han, Gen-Yue Li, Kai-Li Yang, Yuan Zhao, Jia-Qi Wang, Yang-Bin Lai, Chang-Bao Chen, En-Peng Wang

**Affiliations:** Jilin Ginseng Academy, Changchun University of Chinese Medicine, Changchun 130117, China

**Keywords:** red ginseng, by-product, repeated thermal treatment, frequency range

## Abstract

Steamed ginseng water (SGW) is a by-product of the repeated thermal processing of red ginseng, which is characterized by a high bioactive content, better skin care activity, and a large output. However, its value has been ignored, resulting in environmental pollution and resource waste. In this study, UHPLC-Q-Exactive-MS/MS liquid chromatography–mass spectrometry and multivariate statistical analysis were conducted to characterize the compositional features of the repeated thermal-treated SGW. The antioxidant activity (DPPH, ABTS, FRAP, and OH) and chemical composition (total sugars, total saponins, and reducing and non-reducing sugars) were comprehensively evaluated based on the entropy weighting method. Four comparison groups (groups 1 and 3, groups 1 and 5, groups 1 and 7, and groups 1 and 9) were screened for 37 important common difference markers using OPLS-DA analysis. The entropy weight method was used to analyze the weights of the indicators; the seventh SGW sample was reported to have a significant weight. The results of this study suggest that heat treatment time and frequency can be an important indicator value for the quality control of SGW cycling operations, which have great potential in antioxidant products.

## 1. Introduction

*Panax ginseng* C. A. Mey., which belongs to the Araliaceous family, is a perennial herbaceous plant and is one of the most well-known Chinese folk medicines [1]. According to the different processing methods, ginseng products can be classified into different categories, including sundew ginseng, Dali ginseng, red ginseng, and black ginseng [2,3]. In recent years, it has been reported that nearly 13,000 tons of fresh ginseng is processed into red ginseng in Jilin Province, China, which is the most well-known ginseng producing district, producing 1/3 of its global output [4,5,6]. SGW is the main liquid by-product that occurs during the red ginseng thermal manufacturing process; it has a light-yellow color and a strong ginseng flavor, and is accumulated during the evaporating process [7,8]. In Chinese folklore, people usually gather the SGW as a natural anti-aging, whitening, and anti-wrinkling skincare material; however, it is often discarded as a waste material during industrial processing, causing environment pollution and resource waste.

An excessive exposure to solar UV irradiation and oxidizing free radicals could lead to the aging of human skin. For a long time, people have been looking for natural and effective skincare materials. Ginseng, as the most popular botanical cosmetic material, was found to have better skincare activities after the appropriate heat treatment [9,10]. During the evaporating process, hydrolysis, isomerization, and decarboxylation degradation reactions could occur either simultaneously or separately; for instance, the major ginsenosides, which are the most functional ingredients in ginseng, could be converted into rare ginsenosides. As such, the characteristic activities of ginseng are thus improving [11]. In addition, many sesquiterpenes, including sesquiterpene polyacetylene and the products of the Maillard reaction, are found in steamed ginseng water extracts; other types of glycosides, like phenolics, lignans, amino acids, polysaccharides, and aliphatic and aromatic compounds, were also found in the water extract [12]. The origin of the effective utilization of SGW resources is still unclear, and few documents have reported on the impact of repeated thermal processes on the variation in the composition and antioxidant activities of SGW.

Although red ginseng has a high medicinal value, it is necessary to determine an alternative that is has a high yield and a low cost. As the heat treatment time was extended, the chemical composition and activity of SGW changed dynamically. In the current study, we investigated the variation in the content of SGW when it was subjected to a repeated thermal process from 1 to 9 times; this was carried out through the observation on the total sugar, total saponins, and reducing sugar contents. By using UHPLC-Q-Exactive-MS/MS liquid mass spectrometry, the chemical constituents of SGW were identified when combined with the principal component analysis (PCA) and orthogonal partial least squares discriminant analysis (OPLS-DA) methodologies. A total of eight ginsenosides that shared Q-markers were identified by comparing the four model groups of SGW (group 1 and 3, groups 1 and 5, groups 1 and 7, and groups 1 and 9). Thermal processing not only has effects on SGW’s chemical composition, but also on its antioxidant activities. To evaluate the trends in the antioxidant activities SGW that has been subjected to between 1 and 9 times repeated thermal processes, the DPPH, ABTS, FRAP reducing capacity, and hydroxyl radicals were monitored. Our findings showed a scientific method for effectively using the by-products of red ginseng’s repeated thermal processing, as well as a promising way to provide a theoretical basis for its potential on antioxidant products.

## 2. Results

### 2.1. Plotting of Standard Curves

For calculating the FRAP reduction ability, the following regression equation was used: Y = 3.762 x + 0.1627 (*r* = 0.9994). The antioxidant capacity of the samples was assayed using Fe^2+^ to generate a blue-violet complex with TPTZ under acidic conditions.

For calculating the total sugar content, the following regression equation was used: Y = 3.456 x + 0.128 (*r* = 0.9958). The absorbance values were substituted into the standard curve as y-values, and the x-values were calculated to obtain the total sugar content.

For calculating the reducing sugar content, the following regression equation was used: Y = 1.1634 x − 0.0404 (*r* = 0.9936). The Y value is the absorbance value of SGW, while the X value is calculated using the formula to obtain the reducing sugar content.

For calculating the total saponins content, the following regression equation was used: Y = 0.5801 x + 0.0196 (*r* = 0.9995). The Y value gives the absorbance value of SGW, and the X value is calculated using the formula to obtain the total saponins content.

### 2.2. Quantitative Determination of SGW

**Figure 1:**
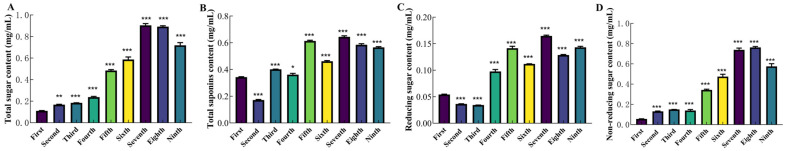
Four different types of content of steamed ginseng water (SGW) subjected to between 1 and 9 times of repeated thermal treatments. (**A**) Total sugar content of SGW. (**B**) Total saponins content of SGW. (**C**) Total reducing sugar content of SGW. (**D**) Total non-reducing sugar content of SGW. (* *p* < 0.05; ** *p* < 0.01; *** *p* < 0.001 vs. SGW1).

The time and frequency of the repeated thermal processing affected the total sugar content. As shown in Figure 1, the highest total sugar content of the seventh SGW was 0.90 mg/mL. The total saponin content of ginseng showed a fluctuating trend during repeated heat treatment, and the highest total saponin content of the seventh SGW was 0.64 mg/mL. The highest content of reducing sugars was 0.16 mg/mL in the seventh SGW, while in the eighth SGW, this value was 0.76 mg/mL.

### 2.3. Results of Chemical Composition 

#### 2.3.1. Multivariate Statistical Analysis of Chemical Composition following Repeated Thermal Processes on SGW

**Figure 2:**
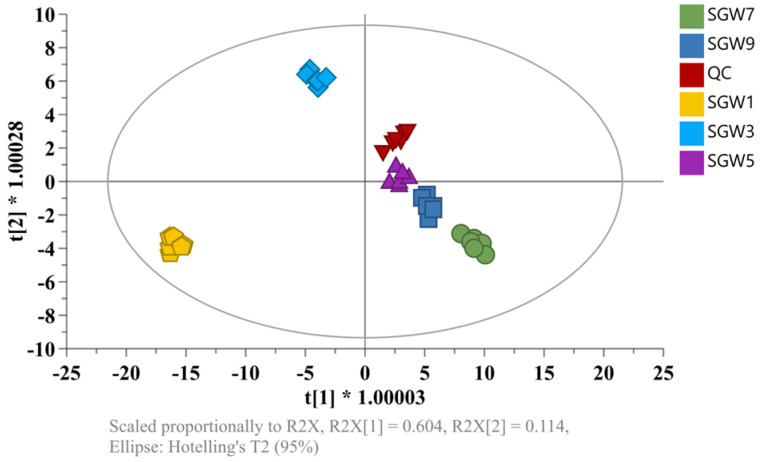
Principal component analysis (PCA) scores of the SGW and QC samples in the (−) negative ion mode.

PCA is a different model, which is based on projection, to provide a path for the data observation from an informatic point of view [13]. The different SGWs were analyzed using UHPLC-Q-Exactive-MS/MS. The extraction and optimization of peaks and the alignment of retention times were performed on LC-MS data using XCMS. PCA analysis was performed in unsupervised mode to clearly differentiate the SGW samples to provide a more intuitive and fine-grained view of the group differences. As shown in Figure 2, SGW was clearly separated into six groups. The quality control (QC) samples were tightly clustered, which indicated that the established PCA model had a good fit and predictability.

#### 2.3.2. Discovery and Identification of Biomarkers for Chemical Composition of Repeated Thermal Processes on SGW

**Figure 3:**
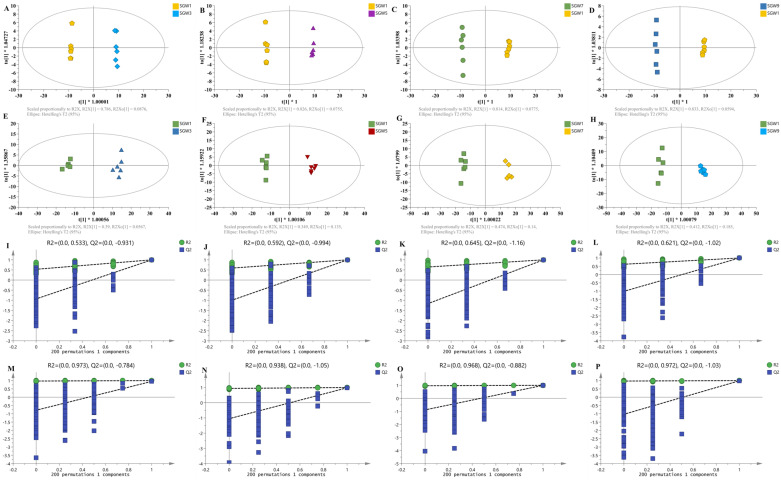
Multivariate statistical analysis base on components from steaming ginseng water with 1-,3-,5-,7-,9-SGW. (**A**–**D**) OPLS-DA score plots in the (−) negative ion mode. (**E**–**H**) OPLS-DA score plots in the (+) positive ion mode. (**I**–**L**) Permutation test in the (−) negative ion mode. (**M**–**P**) Permutation test in the (+) positive ion mode.

**Figure 4:**
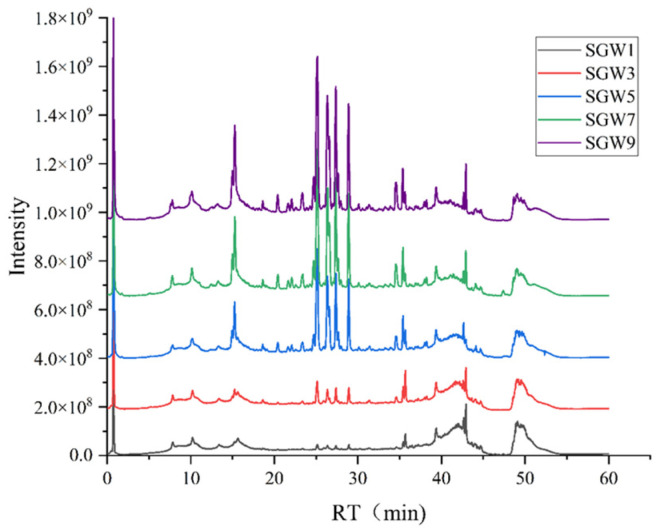
The total ion chromatograms (TICs) of SGW samples after different repeated thermal processes using UHPLC-Q-Exactive-MS/MS in the negative ion mode.

The OPLS-DA model was utilized to generate the score plots, from which it can be seen that the model has a good predictive ability (Figure 3). Additional validation was then performed using a permutation test (200 times) to assess the validity of the OPLS-DA model. All R^2^ (cum) and Q^2^ (cum) values calculated from the data for each mutation were lower than the original values of the developed model, and the inner cross section of the regression line for Q^2^ on the y-axis was negative; this indicates that the developed model was not overfitted and had a good fitting and predictive ability. Based on the importance values of the projected variables (VIP) in the OPLS-DA model, combined with the *t*-test results, potential candidate biomarkers in the first, third, fifth, seventh, and ninth evaporated ginseng water samples were screened out, according to a VIP > 1 and a *p* < 0.05. The results showed that 330 differential compounds were screened in the first vs. third ratio; a total of 250 differential compounds were screened in the first vs. fifth ratio; in total, 289 differential compounds were screened in the first vs. seventh ratio; a total of 250 differential compounds were screened in the first vs. ninth ratio. Based on the above screened compounds, 37 differential compounds were screened with FC > 2 and *p* < 0.01. The total ion chromatograms (TICs) of the SGW samples after different repeated thermal processes using UHPLC-Q-Exactive-MS/MS are shown in Figure 4. The results of the identification of the differential components are shown in Table 1.

### 2.4. Radical Scavenging Activity

**Figure 5:**
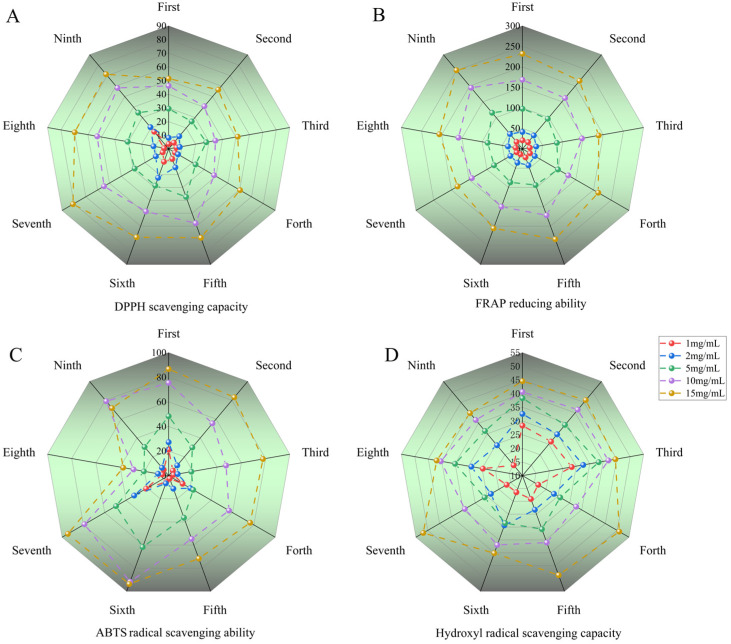
Comparison of antioxidant activities of all SGW samples, which were subjected to different heating times. (**A**) DPPH radical scavenging ability. (**B**) FRAP reducing ability. (**C**) ABTS radical scavenging ability. (**D**) OH scavenging ability.

The free radical scavenging capacity was evaluated according to the IC_50_ value and the scavenging percentage (the IC_50_ value is defined as the concentration of the antioxidant that is needed to scavenge 50% of the free radicals present in the test solution). Various methods were used to evaluate the antioxidant activity of repeatedly heat-processed SGW. The results of the DPPH/ABTS/FRAP/OH antioxidant indices showed that the antioxidant capacity of each group of SGW samples increased in a concentration-dependent manner in the range of 1–15 mg/mL, as shown in Figure 5. The smaller the IC_50_ value, the higher the antioxidant capacity [14]. The ABTS and hydroxyl radical scavenging capacity (IC_50_) of seventh SGW sample were 3.74 mg/mL and 21.48 mg/mL, respectively. In the DPPH antioxidant assay, the seventh SGW sample reported a value of 7.78 mg/mL, which was second only to the fifth and ninth samples. The results of the IC_50_ values of the antioxidant capacity of all the SGW samples are shown in Table 2.

### 2.5. Entropy Weight Method to Analyze SGW Weights

The principle of the entropy weighting method is to analyze the size of the information reflected by different indicators and to assign corresponding weights to these indicators, to make the evaluation results closer to the actual situation. The entropy weight method effectively eliminates the interference of subjective factors, making the decision-making process more objective and accurate. Entropy is an indicator that measures the degree of disorder or chaos in a system. The smaller the entropy value of the indicator, the greater the amount of information it contains, the greater its role in the comprehensive evaluation, and the larger its weight should be. Total sugar, total saponin, reducing sugar, non-reducing sugar, OH, ABTS, FRAP, and DPPH were assigned weights using the entropy weighting method. As shown in Table 3 and Table 4, the weights of the different SGW samples that were subjected to different repeated thermal processing treatments were compared by composite values. The following was found: seventh SGW > ninth SGW > fifth SGW > eighth SGW > sixth SGW > fourth SGW > second SGW > first SGW > third SGW. The specific steps are as follows:

Step 1: Standardize the raw data. Due to the different attributes of the evaluation indicators, it is necessary to properly standardize the raw data in order to eliminate the effect of dimensional differences and to ensure the comparability of data and the feasibility of the decision-making results [15]. As such, the following is obtained:

$$\frac{Y_{ij}=(X_{ij}-min\left\{X_{ij}\right\})}{max\left\{X_{ij}\right\}-min\left\{X_{ij}\right\}}$$

Step 2: Normalization of standardized data.

$$f_{ij}=\frac{Y_{ij}}{\sum_{i=1}^{n}Y_{ij}}$$

Step 3: Information entropy of each indicator.

$$\underset{f_{\mathrm{ij}}\to 0}{\lim}f_{ij}\ln f_{ij}=0$$

Step 4: Confirmation of the weights of the indicators.

$$W_{j}=\frac{\left(1-E_{j}\right)}{\left(m-\sum_{j=1}^{m}E_{j})(j=1,2,\ldots m\right)}$$

Step 5: Synthesize value.

$$V_{i}=\sum_{j=1}^{n}(W_{j}\times Y_{ij})(i=1,2,\ldots m)$$

## 3. Discussion

In this study, the quantitative determination, chemical composition, and antioxidant activity of different SGW samples after repeated heat treatments were investigated. The results showed that SGW with an optimal selection of heating time and number of process repeats can be used for antioxidant product development and industrial raw material preparation. After repeated heat treatments, the chemical composition content was found to be closely related to the heat treatment method, the temperature, and the time [16,17,18]. The contents of total sugars, total saponins, and reducing sugars were highest at the seventh heat treatment. Thermal instability degraded ginsenosides; this was responsible for the decrease in ginsenoside content in the second heat treatment. Polar ginsenosides were not completely degraded and some ginsenosides were degraded to produce secondary ginsenosides following the second heat treatment, which was the reason for the increase in ginsenoside content in the third heat treatment [19]. The content of ginsenosides was highest at the seventh treatment and gradually decreased at the eighth and ninth treatments, as a results of over-processing. At the initial stage of ginseng heating, malonyl ginsenoside side chain molecules are unstable and hydrolyzed under the action of acid, alkali, or high temperatures, subsequently converting to neutral saponins [20]. In addition, polar ginsenosides Rg1/Re, Rc, Rb2, and Rd were gradually reduced and rare ginsenosides Rg2, Rg3, F4, Rg5, Rk1, Rh2, Rh1, and Rh4 were gradually generated, which enhanced the antioxidant activity during heat treatment [21,22,23,24].

Reducing sugars mainly include glucose, fructose, galactose, and maltose. After repeated heat treatment, ginsenosides are hydrolyzed to glycosides and sugars [25]. Non-reducing sugars mainly include starch, sucrose, and arabinogalactan. SGW was continuously reduced and concentrated by repeated rounds of heating [26]. Thermal processing leads to a decrease in the amount of water-soluble polysaccharides and an increase in the amount of acidic polysaccharides [27]. Starch was the main component of ginseng polysaccharides that were extracted from ginseng roots. During repeated heating and steaming processes, ginseng starch was pasted to form monosaccharides or oligosaccharides. The viscosity of ginseng starch gradually increased after the seventh and eighth steaming cycle [28].

According to related studies, the C-20 sugar in the side chain of ginsenoside was hydrolyzed first, followed by the C-6 or C-3 sugars. In addition, C-20 stereoisomerization was also an important structural change [29,30]. Using UHPLC-Q-Exactive-MS/MS liquid mass spectrometry analysis, 37 chemical components of SGW were identified, including ginsenosides Re, Rb1, Rb2, Rc, Rd, and Ro. Multivariate statistical analysis revealed that the first SGW and the third, fifth, seventh, and ninth SGW were well separated. The quality control samples were clustered with a large contribution of SGW1, and the method was stable and predictive. The entropy weight method is an objective assignment method, which is objective and credible; it was applied to determine the weights of content and antioxidant indexes, and the combined values were analyzed in the seventh SGW, which had the largest weight. The heat treatment of ginseng produces maltol, salicylic acid, vanillic acid, and p-coumaric acid, which scavenge free radicals and enhance antioxidant activity [31,32,33]. In addition, our research group compared the seventh SGW with steamed red ginseng and found that the total saponins content could be up to 63.4% that of red ginseng. In the future, the comprehensive utilization of SGW could provide a potential raw material for the development of antioxidant products, which, on the one hand, can reduce the waste of ginseng resources and improve the economic benefits, and, on the other hand, can protect the environment and help towards sustainable development.

## 4. Materials and Methods

### 4.1. Materials

Fresh roots of 5-year-old ginseng were purchased from Wanliang Town, Fusong County, Jilin Province, and were identified by Professor Weng Lili from Changchun University of Traditional Chinese Medicine. UPLC-grade acetonitrile and methanol were obtained from Tedia Company Incorporated (Fairfield, OH, USA). Purified water was made using a water purifier (Global Water Solution Ltd., Randolph, MA, USA). Ginsenoside Re was purchased from Shanghai yuanye Bio-Technology Co., Ltd. (Shanghai, China).

### 4.2. Extraction and Preparation of Steamed Ginseng Extract

Fresh ginseng was washed and neatly arranged with the reeds facing down. The ginseng was steamed with 2000 mL of distilled water at 100 °C for 2 h. At the end of steaming, the water was replenished to 2000 mL. Meanwhile, the process was repeated 9 times by replacing fresh ginseng of the same size and quality. The 9 groups of SGW treated by repeated steaming were concentrated to 200 mL under a vacuum pressure of minus 0.08 Mpa at 55 °C. Then, the samples were centrifuged at 1000× *g* for 5 min and the supernatant was freeze-dried and kept as a spare.

### 4.3. Sample Preparation for Mass Spectrometry

A total of 5 mg SGW lyophilized powder was accurately weighed and dissolved in 50 mL methanol to make a concentration of 0.1 mg/mL; a 10 µL sample solution was added into 0.99 mL methanol in a 2.5 mL Eppendorf tube, vibrated for 30 s, and then filtered using a 0.22 μM PTFE membrane; this was then refrigerated at 4 °C and kept as a spare.

### 4.4. The Quantitative Analysis of SGW

#### 4.4.1. Total Sugar Content Assay

Glucose was accurately weighed and dried in a constant-weight oven at 105 °C. A glucose solution of 0.1 mg/mL was prepared. Then, 2 mL glucose diluent of different concentrations, 1 mL 5% phenol solution, and 5 mL sulfuric acid concentrated solution were added [34]. A water bath was heated at 40 °C for 30 min and was then cooled to room temperature. The absorbance was measured at 490 nm, and a standard curve was drawn. The glucose content of the SGW samples of different times was calculated using regression equations.

#### 4.4.2. Total Saponins Content Assay

The activated D101 macroporous adsorption resin column was prepared and activated again with ethanol and distilled water. First, different repeated heat treatment SGW samples (1 mL) and 25 mL distilled water for the washing column were precisely added twice, and the eluent was discarded. Then, the ginsenosides were slowly washed off with 25 mL 70% ethanol, and the eluent was collected in the evaporating dish. Finally, the water bath evaporated and dried at 60 °C.

A ginsenoside Re standard diluted to 1 mg/mL was prepared. First, it was placed into a solution of 0 mg/mL, 0.02 mg/mL, 0.04 mg/mL, 0.06 mg/mL, 0.08 mg/mL, and 1 mg/mL in a tube with plugs. Then, 0.2 mL of vanillin and 0.8 mL of perchloric acid were added to the solution. Finally, the water bath was heated at 60 °C for 10min. After cooling, glacial acetic acid was added at a constant volume to 5.0 mL. The ginsenoside content of SGW samples was calculated by substituting the absorbance value into the standard curve.

#### 4.4.3. Total Reducing Sugar Content Assay

The reducing sugar content in SGW samples was determined using a reducing sugar kit and spectrophotometry. SGW samples subjected to different numbers of repeats pf thermal processing (100 μL) and 100 μL reagent 1 (colorimetric solution) were thoroughly mixed in a centrifuge tube. Next, a water bath was heated at 95 °C for 10 min, before being cooled. The control group was replaced with distilled water, and the absorbance was measured at 500 nm.

### 4.5. UHPLC-Q-Exactive-MS/MS Conditions

Chromatographic conditions: Separation was performed using an Ascentis^®^ Express C_18_ (3.0 mm × 500 mm, 2.7 μm; Supelco, Bellefonte, PA, USA). The mobile phase consisted of 0.1% formic acid (solvent A) and acetonitrile (solvent B). The gradient elution program was as follows: 0–3 min 25% B, 3–20 min 25–32% B, 20–55 min 32–40% B, 55–56 min 40–95% B, and 56–60 min 95–25% B; the flow rate was set at 0.3 mL/min. The column and sampler temperatures were set at 35 °C and 4 °C, respectively. The injection volume was 5 μL.

Mass spectrometry conditions: An UHPLC-Q-Exactive-MS/MS (Thermo Fisher Scientific, Waltham, MA, USA) equipped with an Xcalibur data acquisition system and electrospray ionization source were used in both the positive and negative ion modes. Electrospray ion source (ESI), positive and negative ion mode detection, scanning type: Full scan-ddMS^2^. Mass scanning range: 150.0–2000.0 *m*/*z*. The flow rate of the sheath gas was 40 arb, the aux gas was 10 arb, the spray voltage was (±) 3.5 KV, the capillary temperature was 350 °C, the fragmentation voltage was 30 ev, and the mass axis was calibrated before detection.

### 4.6. Antioxidant Activity Assays

#### 4.6.1. DPPH Scavenging Activity Assay

The DPPH scavenging activity was assayed according to a previous study, with a few minor adjustments [35]. The method involves the repeated thermal processing of SGW with 0.2 mM of DPPH alcohol solution. Briefly, 80 µL of diluted DPPH solution and 50 µL of sample solutions were mixed in a microplate and were incubated for 30 min in the dark. The absorbance of the sample was recorded at 520 nm.

#### 4.6.2. FRAP Scavenging Activity Assay

The FRAP solution was obtained by mixing sodium acetate buffer (pH 3.6, 0.3 mol/L), TPTZ solution (10 mmol/L), and FeCl_3_ solution (20 mmol/L) at a ratio of 10:1:1 [36,37]. Briefly, 200 µL of diluted FRAP solution and 50 µL of sample solutions were mixed in a microplate and incubated for 10 min at 37 °C. The absorbance of the sample was recorded at 592 nm. The antioxidant capacity is expressed as the mass concentration of ferrous sulfate (mg/L), using ferrous sulfate solution as the standard curve.

#### 4.6.3. ABTS^+^ Scavenging Activity Assay

A solution of 7.0 mM ABTS^+^ and 2.45 mM potassium persulfate was mixed evenly in equal proportions and stored in the dark for 12–16 h [38,39]. The absorbance of the diluent at 734 nm was 0.70 ± 0.02. The sample solution of 50 μL was added into the diluted ABTS^+^ solution of 80 μL and was reacted in the dark for 6 min, before being measured at 734 nm.

#### 4.6.4. Hydroxyl Radical Assay

The hydroxyl radical assay was performed according to a previous study, with minor optimization [40,41]. A solution of 5.0 mM FeSO_4_, 5.0 mM ethanolic salicylic acid, and 3 mM H_2_O_2_ was mixed and was reacted for 30 min at 37 °C. The absorbance of the sample was recorded at 510 nm.

### 4.7. Data Processing and Multivariate Analysis

SPSS 23.0 (SPSS Inc., Chicago, IL, USA) was used for one-way ANOVA, and Bonferroni was used for significance testing to analyze the significant differences (*p* < 0.05) in the peak areas of the chemical composition in four groups of SGW. Statistical analysis was performed from triplicate results and the mean and standard deviation (mean ± SD) were listed.

All the original MS spectrometry data were preprocessed and exported. Then, retention time calibration, peak alignment, baseline calibration, normalization, and logarithmic conversion were conducted. Multivariate analyses were performed using SIMCA-P software 17.0 (Umetrics, Umea, Sweden). The datasets of peak areas were scaled (UV or Pareto) prior to multivariate analysis using PCA or OPLS-DA. The variable importance in the projection (VIP) values indicate the major compounds contributing to the separation of each sample in the OPLS-DA score plots. The VIP value is a weighted sum of squares of the OPLS-DA weights that takes the explained Y variance in each dimension into account. The OPLS-DA models were validated with permutation tests. The biomarkers were analyzed using MS/MS analysis and the metabolite database HMDB (http://www.hmdb.ca/).

## 5. Conclusions

SGW, as a valuable resource that is produced during the red ginseng manufacturing processing, is widely used for skin care and health enhancement in folklore of the Changbai mountain region in the northeast of Jilin province, China. Since its composition and activity are unclear, the scientific basis for being recycled is insufficient. In the present study, we investigated the correlation between the frequency of sample reheating and skincare properties, including anti-aging effects and composition content levels. The results showed that the number of heating cycles significantly impacts the content of saponins and sugars (including polysaccharides, reducing sugars, and non-reducing sugars). The results of the frequency of thermal heating and compositional analyses proved that the process of generating SGW, if the maintenance of its antioxidant activity is desired, is minimized following its repeated heating process; it is recommended not to exceed seven cycles. In summary, this study may provide a solid scientific proof as to the high-quality recycling of red ginseng-processed by-products, and may deepen the application value of SGW in the antioxidant industry.

## Figures and Tables

**Table 1:** Identification of shared differential ginsenoside compounds using UHPLC-Q-Exactive-MS/MS analysis.

Pattern	No.	Extract *m*/*z*	t/min	Metabolites	Formula	Monoisotopic Mass	Adduct	Mass Error (mDa)	1 vs. 3	1 vs. 5	1 vs. 7	1 vs. 9
ESI^−^	1	435.0563	0.82	3,4-Dihydroxyphenylpyruvate	C_15_H_20_O_10_	195.0299	2M+FA-H	4	−1.59	−2.21	−2.49	−2.42
	2	777.1628	1.16	3-Methylbutyl glucosinolate	C_6_H_12_N_3_PS	389.0814	2M-H	9	−2.36	−3.26	−3.54	−3.32
	3	779.1603	1.16	Dimethyl 2-galloylgalactarate	C_30_H_40_O_6_	390.0798	2M-H	10	−2.30	−3.20	−3.47	−3.28
	4	719.2043	1.17	3-Methoxy-4-hydroxyphenylglycol glucuronide	C_54_H_92_O_23_	360.1056	2M-H	0	−2.06	−3.02	−3.30	−2.96
	5	539.14	1.24	Phenylglucuronide	C_29_H_40_N_8_O_5_	270.0740	2M-H	1	−1.28	−1.97	−2.21	−1.99
	6	767.5313	1.26	Persicachrome	C_58_H_98_O_26_	384.2664	2M-H	7	0.26	0.83	1.58	1.49
	7	387.1176	5.84	Ferulic acid	C_54_H_92_O_24_	194.0579	2M-H	23	4.14	1.60	3.44	3.61
	8	455.1047	5.84	Epicatechin 3-O-(4-methylgallate)	C_53_H_90_O_22_	456.1056	M-H	14	8.87	1.94	7.44	7.80
	9	767.5309	5.88	Persicaxanthin	C_48_H_82_O_18_	384.2664	2M-H	7	1.34	1.32	2.11	1.69
	10	377.0881	5.94	3,3′,5-Trihydroxy-4′,7-dimethoxyflavanone	C_42_H_66_O_14_	332.0896	M+FA-H	1	7.14	1.30	6.45	6.64
	11	991.5515	15.38	Ginsenoside Re	C_10_H_10_O_4_	946.5501	M+FA-H	3	−2.28	−3.39	−3.99	−3.67
	12	815.4829	21.62	Majonoside R1	C_12_H_14_O_7_	816.4871	M-H	4	−2.01	−3.27	−4.01	−3.62
	13	1107.596	24.96	Ginsenoside Rb1	C_12_H_23_NO_9_S_2_	1108.6029	M-H	0	−2.70	−4.30	−4.96	−4.70
	14	955.4933	26.04	Ginsenoside Ro	C_29_H_46_O_3_	956.4981	M-H	3	−1.87	−2.80	−3.01	−1.89
	15	561.293	26.23	Hordatine B	C_15_H_18_O_12_	580.3122	M-H_2_O-H	1	−2.87	−4.20	−4.77	−4.51
	16	1209.629	26.43	Ginsenoside Ra1	C_25_H_36_O_3_	1210.6346	M-H	1	−2.73	−4.30	−4.85	−4.41
	17	1123.594	27.05	Ginsenoside Rb2	C_23_H_20_O_10_	1078.5924	M+FA-H	3	−2.32	−3.63	−4.23	−3.97
	18	1077.584	27.24	Ginsenoside Rc	C_25_H_36_O_3_	1078.5924	M+FA-H	3	−2.50	−4.02	−4.75	−4.42
	19	945.5425	28.76	Ginsenoside Rd	C_12_H_22_O_11_	946.5501	M-H	0	−2.37	−3.98	−4.76	−4.37
	20	793.4408	34.44	Spinasaponin A	C_42_H_66_O_14_	794.4453	M-H	4	−2.35	−3.45	−4.38	−4.34
	21	333.2318	42.87	(S)-10,16-Dihydroxyhexadecanoic acid	C_16_H_32_O_4_	288.2301	M+FA-H	11	−0.62	3.28	0.17	0.95
	22	369.2081	42.87	Ecgonine	C_9_H_15_NO_3_	185.1052	2M-H	14	−0.46	2.85	0.13	0.74
	23	423.33	44.04	Camellenodiol	C_29_H_46_O_3_	442.3447	M-H_2_O-H	9	−1.05	2.79	−0.02	1.48
ESI^+^	24	462.3461	1.39	Galactosylsphingosine	C_24_H_47_NO_7_	461.3353	M+H	8	0.42	0.89	1.14	1.13
	25	367.2169	1.44	Demethoxyfumitremorgin C	C_21_H_23_N_3_O_2_	349.1790	M+NH_4_	11	0.00	0.24	0.69	0.43
	26	300.2	1.92	Miltirone	C_19_H_22_O_2_	282.1620	M+NH_4_	14	−0.55	2.29	0.65	0.23
	27	328.2313	1.97	Menaquinol	C_21_H_26_O_2_	310.1933	M+NH_4_	13	−0.35	2.68	1.02	0.63
	28	344.2258	2.12	Isopiperolein B	C_19_H_30_O_5_	343.2147	M+H	11	−0.46	2.65	0.69	0.24
	29	327.2	2.18	Heptaethylene glycol	C_14_H_30_O_8_	326.1941	M+H	6	−0.50	2.60	0.67	0.20
	30	388.2518	2.44	Octaethylene glycol	C_16_H_34_O_9_	370.2203	M+NH_4_	6	−0.43	2.92	0.77	0.30
	31	460.3084	2.85	Muzanzagenin	C_27_H_38_O_5_	442.2719	M+NH_4_	4	−0.41	3.55	0.85	0.29
	32	476.3039	3.34	Lucidenic acid A	C_27_H_38_O_6_	458.2668	M+NH_4_	5	−0.46	3.14	0.83	0.32
	33	605.3827	3.92	Ginsenoyne H	C_19_H_26_O_3_	302.1882	2M+H	2	1.06	−0.45	2.17	1.04
	34	567.4277	3.92	Cryptocapsone	C_40_H_54_O_2_	566.4124	M+H	14	0.92	−0.18	1.35	0.96
	35	679.5078	3.92	Avocadene 1-acetate	C_19_H_36_O_4_	328.2614	2M+Na	6	−0.17	0.13	−0.11	−0.03
	36	358.2571	43.05	2-alpha-Ethoxydihydrophytuberin	C_19_H_32_O_5_	340.2250	M+NH_4_	5	−0.13	−0.01	−0.21	0.09
	37	359.2593	43.16	Isolinderanolide	C_21_H_36_O_3_	336.2664	M+Na	10	0.07	0.09	0.02	0.09

Note: 1 vs. 3, 1 vs. 5, 1 vs. 7, and 1 vs. 9 mean Log _2_ (fold change).

**Table 2:** Statistical analysis of SGW samples (IC_50_).

IC_50_	SGW1	SGW2	SGW3	SGW4	SGW5	SGW6	SGW7	SGW8	SGW9
DPPH	12.64	12.74	15.33	12.27	7.78	9.15	8.10	8.82	7.34
FRAP	2.08	2.04	2.34	2.24	2.05	2.31	2.41	2.29	1.93
ABTS^+^	4.28	7.84	9.67	8.08	8.10	4.33	3.74	27.27	6.86
OH	38.43	28.54	34.09	22.6	22.81	43.69	20.33	41.32	35.46

**Table 3:** The entropy weighting method analyzes the weights of the indicators.

Layers	Indicator	Weight
Contents	Total sugar	0.179324
	Total saponins	0.081962
	Reducing sugar	0.149848
	Non-reducing sugar	0.173238
Antioxidant capacities	OH	0.134299
	ABTS	0.056045
	FRAP	0.132912
	DPPH	0.092371

**Table 4:** Entropy weighting method to analyze the synthesize value of different SGW samples.

Sample	Comprehensive Value
SGW1	0.260027031
SGW2	0.299462879
SGW3	0.196067887
SGW4	0.404293176
SGW5	0.707125369
SGW6	0.50339906
SGW7	0.852184669
SGW8	0.652176659
SGW9	0.779304053

## Data Availability

The datasets used and/or analyzed in the present study are available from the corresponding author upon reasonable request.
